# γδ T17 Cells Regulate the Acute Antiviral Response of NK Cells in HSV-1–Infected Corneas

**DOI:** 10.1167/iovs.65.13.16

**Published:** 2024-11-06

**Authors:** Rachel R. Rodenberg, Domenico Spadafora, Steffani Fitzpatrick, Grant Daly, Robert Lausch, Robert A. Barrington

**Affiliations:** 1 Department of Microbiology & Immunology, University of South Alabama, Mobile, Alabama, United States; 2 Flow Cytometry Shared Resources Laboratory, University of South Alabama, Mobile, Alabama, United States; 3 Department of Pharmacology, University of South Alabama, Mobile, Alabama, United States

**Keywords:** γδ T cells, interleukin 17A, natural killer cells, interferon γ, granzyme, herpes simplex virus 1, corneal opacity

## Abstract

**Purpose:**

To determine whether γδ T cells regulate natural killer (NK) cells in the herpes simplex virus 1 (HSV-1)–infected cornea.

**Methods:**

CD57Bl/6 (wild-type [WT]), TCRδ^−/−^, and IFN-γ^−/−^ mice were infected intracorneally with HSV-1. TCR^−/−^ mice were treated with IL-17A at 24 hours post-infection (PI), and the WT mice received treatments of fingolimod (FTY720) and anti-IL-17A. At 48 hours PI, corneas were excised, and intracellular staining flow cytometry was performed, as well as multiplex analysis. Additionally, single-cell RNA sequencing (scRNAseq) was done to analyze the transcriptome of NK cells from WT and TCRδ^−/−^ mice.

**Results:**

In mice lacking γδ T cells, there were significantly fewer NK cells following ocular HSV-1 infection. This reduction of NK cells corresponded with lower levels of cytokines and chemokines associated with the antiviral response. Furthermore, NK cells from WT mice had enriched IL-17A signaling compared to those from TCRδ^−/−^ mice. The NK cell response was partially rescued in TCRδ^−/−^ mice by administration of IL-17A. Correspondingly, the NK cell response could be blunted in WT mice by administration of anti–IL-17A. Finally, IFN-γ^−/−^ mice had significantly less IL-17A production compared to WT mice.

**Conclusions:**

γδ T17 cells promote NK cell accumulation in HSV-1–infected corneas. In turn, NK cells secrete IFN-γ, which negatively regulates further IL-17A production by γδ T cells.

Herpes stromal keratitis (HSK), caused by herpes simplex virus 1 (HSV-1) infecting the cornea, is the leading cause of infectious blindness in developed countries.^1^ Once established, HSV-1 can reactivate, promoting more corneal damage over time that ultimately results in blindness and, in some cases, herpetic encephalitis.^1^^–^^3^ The standard of care for patients is antiviral therapy, but with the increased incidence of resistant viral strains due to long-term antiviral use, immunotherapeutic strategies are being investigated.^4^^–^^6^

Early innate immune responses to HSV-1 are generally protective, whereas later adaptive immune responses result in marked corneal pathology.^7^^,^^8^ γδ T cells are a critical component of the early response to HSV-1, as mice lacking γδ T cells (TCRδ^−/−^) succumb to HSV-1–induced encephalitis following ocular infection.^3^^,^^9^ Corneal γδ T cells are the major early source of interleukin 17A (IL-17A) during acute infection,^9^^,^^10^ and reconstitution of γδ T17 cells in TCRδ^−/−^ mice is sufficient for protection, suggesting that IL-17A production by γδ T17 cells is necessary for protection against ocular HSV-1.^9^ IL-17A has pleiotropic activities, such as inducing chemokines, antimicrobial peptides, and proinflammatory cytokine production, in addition to aiding in vascular remodeling.^11^ During HSV-1 infection, IL-17A facilitates neutrophil chemotaxis.^9^^,^^12^^,^^13^ Paradoxically, although neutrophils are important in the innate immune response against HSV-1, they contribute to pathogenesis by aggravating corneal damage.^7^^,^^14^ Whether γδ T17 cells and/or IL-17A orchestrate the activation of putative protective pathways, such as targeted killing of virally infected cells, remains unknown.

Antiviral cytotoxic immune responses serve to limit the infectious cycle by direct killing of virus-infected cells.^15^ Natural killer (NK) cells are key effectors of the antiviral response through the secretion IFN-γ and by production of cytotoxic granzymes and perforin.^7^^,^^16^^–^^19^ Although the antiviral function of NK cells is clear, whether NK cells are necessary for protection against ocular HSV-1 infection is unresolved.^20^^–^^22^

In the current study, we demonstrated that IL-17A produced by γδ T17 cells promotes the accumulation of cytotoxic, antiviral NK cells in HSV-1–infected corneas. This was demonstrated in several ways: (1) TCRδ^−/−^ mice had fewer antiviral NK cells following HSV-1 corneal infection compared to wild-type (WT) mice; (2) administration of IL-17A to TCRδ^−/−^ mice restored the NK cell population; and (3) neutralization of IL-17A in the WT mice caused a reduction in NK cells leading to increased viral titers. Additionally, the absence of IFN-γ was associated with enhanced IL-17A production. Collectively, this study identified a novel mechanism that γδ T17 cells employ to provide protection and defined a cross-regulatory pathway between γδ T17 cells and NK cells in the HSV-1–infected cornea.

## Materials and Methods

### Mice

CD57BL/6 (WT), TCRδ^−/−^, and INFγ^−/−^ mice were purchased from The Jackson Laboratory (Bar Harbor, ME, USA). Mice were housed and bred within the University of South Alabama College of Medicine, an Association for Assessment and Accreditation of Laboratory Animal Care (AAALAC)-accredited animal facility. All protocols involving the use of mice were approved by the Institutional Animal Care and Use Committee at the University of South Alabama.

### HSV-1 Infection

Intracorneal infections were performed by injecting HSV-1 strain RE directly into the cornea as previously described.^9^ Briefly, a hole was made in the corneal stroma and 1 µL of HSV-1 was injected into the cornea (1 × 10^6^ plaque-forming units [PFU]) using a repeating dispenser (Hamilton Company, Reno, NV, USA).

### Digestion of Corneal Tissue

Corneal buttons were isolated and transferred with RPMI 1640 Medium (Thermo Fisher Scientific, Waltham, MA, USA) to gentleMACS C Tubes containing digestive enzymes from the Multi Tissue Dissociation Kit 1 (Miltenyi Biotec, Bergisch Gladbach, Germany). Corneas were digested using a modified spleen digest protocol on a gentleMACS Tissue Dissociator. After dissociation, cells were passed through a 70-µm filter and processed for further applications.^23^

### Flow Cytometric Analysis and Intracellular Staining

Single cells were obtained using the corneal digest protocol at 48 hours post-infection (PI). Cells were then processed using an eBioscience Intracellular Fixation & Permeabilization Buffer Set (Thermo Fisher Scientific). Intracellular antibodies specific for mouse granzyme A (GZMA; eBioscience, GzA-3G8.5; Thermo Fisher Scientific), granzyme B (GZMB; eBioscience, 16G6; Thermo Fisher Scientific), and IFN-γ (eBioscience, XMG1.2; Thermo Fisher Scientific) and surface NK1.1 (eBioscience, PK136; Thermo Fisher Scientific), TCRδ (eBioscience, GL3; Thermo Fisher Scientific), Ly6G (eBioscience, 1A8; Thermo Fisher Scientific), CD3ε (eBioscience, 2C11; Thermo Fisher Scientific), and CCR6 (2L17; BioLegend, San Diego, CA, USA) were used. Isotype controls were utilized to confirm staining specificity.

Four corneas were used per sample for flow cytometry analysis. Briefly, viable CD45-positive cells were distinguished from dead or dying cells by forward scatter area (FSC-A) × side scatter area (SSC-A) gating. Single cells were gated based on FSC height (FSC-H) × FSC width (FSC-W), to eliminate potential contributions of cell doublets. γδ T cells were identified as CD45^+^GL3^+^CD3ε^+^ cells, and NK cells were identified as NK1.1^+^CD3ε^neg^ cells. Enumeration of the different corneal cell populations was performed by multiplying the population frequency (as determined by flow cytometry) by the corneal cell number (determined by hemocytometer).

### Single-Cell RNA Sequencing

Single corneal cell suspensions were processed via 10x Genomics Workflow using the Chromium Single Cell 3′ Reagent Kit (10x Genomics, San Francisco, CA, USA). Corneas from 12 mice were pooled and diluted to generate each library. Libraries were sequenced (Novogene Corporation, Sacramento, CA, USA) and the mm10 reference genome was used for analysis. FASTQ files were processed using the Cell Ranger pipeline version 7.0.1, and subsequent library data were visualized and analyzed using Loupe Browser 6.0 (default settings), provided by 10x Genomics (Supplementary Fig. S1). Ten principle components were applied to this dataset. Cells with mitochondrial RNA > 5% were excluded.^24^ Kyoto Encyclopedia of Genes and Genomes (KEGG) pathway analysis was performed via WebGestalt. γδ T cells were identified through the expression of *TRDC*, and NK cells were identified by the expression of *NKG7*.

### Enzyme-Linked Immunosorbent Assay

Enzyme-linked immunosorbent assays (ELISAs) to detect IL-17A and IFN-γ were completed as previously described.^25^ Briefly, capture anti–IFN-γ antibody (XMG1.2; Bio X Cell, Lebanon, NH, USA) was added to each well of a 24-well plate. Corneal cells from WT or IFN-γ^−/−^ mice were added to the plate at various dilutions. After a 24-hour incubation, the plate was washed and a secondary biotinylated anti–IFN-γ (eBioscience, 6A2; Thermo Fisher Scientific) was added.

### Multiplex Analyte Detection Assay

Corneal cell lysates (four per sample) from HSV-1–infected mice were prepared and resuspended in radioimmunoprecipitation assay (RIPA) buffer. Protein concentration was measured using the Bradford assay. Samples were analyzed using a 44-plex cytokine discovery assay (Eve Technologies, Calgary, AB, Canada). Minimal detectable concentrations (pg/mL) were as follows: IFN-γ, 1.1; IL-1α, 10.3; IL-1β, 5.4; IL-6, 1.1; IL-15, 7.4; IL-17A, 0.5; C-X-C motif chemokine ligand 1 (CXCL1), 2.3; macrophage inflammatory protein 1α (MIP-1α), 7.7; MIP-2, 30.6; TNFα, 2.3; vascular endothelial growth factor (VEGF), 0.3; and monokine induced by gamma interferon (MIG), 2.4.

### Administration and Neutralization of IL-17A

At 24 hours PI, TCRδ^−/−^ mice received 50-pg/mL IL-17A intracorneally (IC). At 48 hours PI, corneas were harvested and processed as described above. In separate experiments, WT mice received 100 µL of a 1:10 dilution of 1-mg/mL stock fingolimod (FTY720; Thermo Fisher Scientific), delivered intraperitoneally (IP) at the time of infection. For mice receiving FTY720, IL-17A was administered intracorneally as described above. For neutralization of IL-17A, mice received 200 µg of anti–IL-17A or isotype control (Bio X Cell) at the time of infection. Corneas were harvested at both 48 hours and 7 days PI for further analyses.

### Plaque Assay

Corneas and trigeminal ganglia (TG) were harvested to determine the viral titer by plaque assay using Vero cell lines (American Type Culture Collection, Manassas, VA, USA) as previously described.^9^ Briefly, both tissues were collected in 500 µL of Dulbecco's modified Eagle's medium (DMEM) with 5% fetal bovine serum. Tissues were then homogenized and sonicated on ice. Samples were centrifuged, and supernatants were taken for plaque assay.

## Results

### γδ T Cells Are the Primary Producers of IL-17A in the HSV-1–Infected Cornea

**Figure 1.**
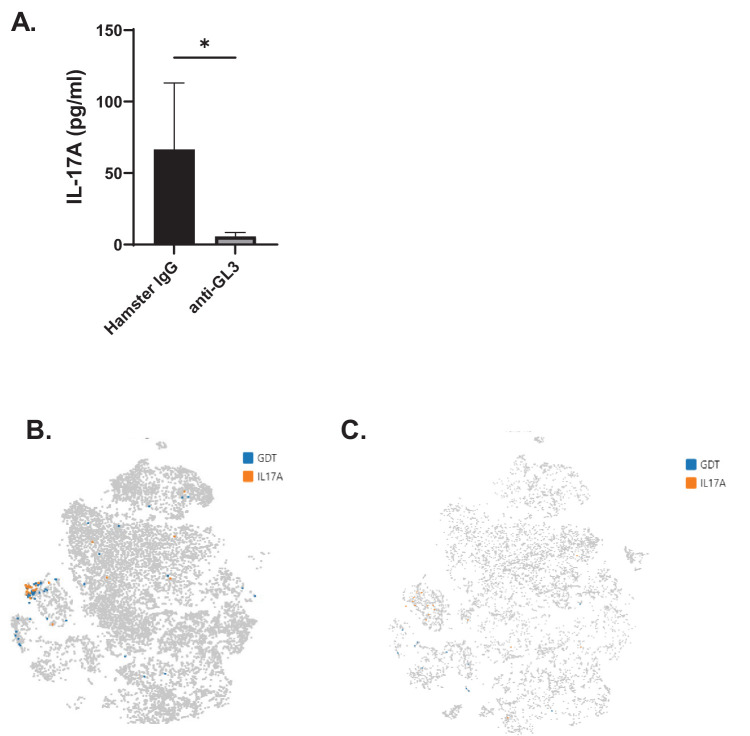
γδ T-cell production of IL-17A during the acute phase of ocular HSV-1 infection. (**A**) ELISA measuring levels of IL-17A from the corneas of HSV-1–infected isotype control mice and mice receiving anti-GL3 (*n* = 3 to 5 mice; data are summarized from two independent experiments). *P < 0.04, unpaired *t*-test. (**B**, **C**) Corneal WT (**B**) and TCRδ^−/−^ (**C**) γδ T cells (*blue*) and IL-17A–expressing cells (*orange*) were compared on *t*-distributed stochastic neighbor embedding (*t*-SNE) plots.

We and others have previously reported that γδ T cells are the primary producers of IL-17A in the HSV-1–infected cornea.^9^^,^^10^ In addition, we previously established that adoptive transfer of γδ T17 cells into HSV-1–infected TCRδ^−/−^ mice was sufficient to provide protection.^9^ As an independent test, γδ T cells were depleted using an anti-GL3 antibody, and IL-17A levels were measured by ELISA and compared to isotype control-treated mice. Corneas from isotype control-treated mice had significantly more IL-17A compared to mice receiving γδ T-cell–depleting anti-GL3 antibody (Fig. 1A). To rigorously evaluate what cells express IL-17A transcript, we performed scRNAseq using corneas from both WT and TCRδ^−/−^ mice. IL-17A was primarily expressed by γδ T cells in the HSV-1–infected cornea by scRNAseq (Fig. 1B). Few IL-17A expressing cells were observed in corneas from TCRδ^−/−^ mice following ocular HSV-1 infection (Fig. 1C). Taken together, these results are consistent with previous studies and further validate that γδ T cells are the primary source of IL-17A during the acute phase of ocular HSV-1 infection.^9^^,^^10^

### HSV-1–Infected Corneas From TCRδ^−/−^ Mice Have an Altered Microenvironment

**Figure 2.**
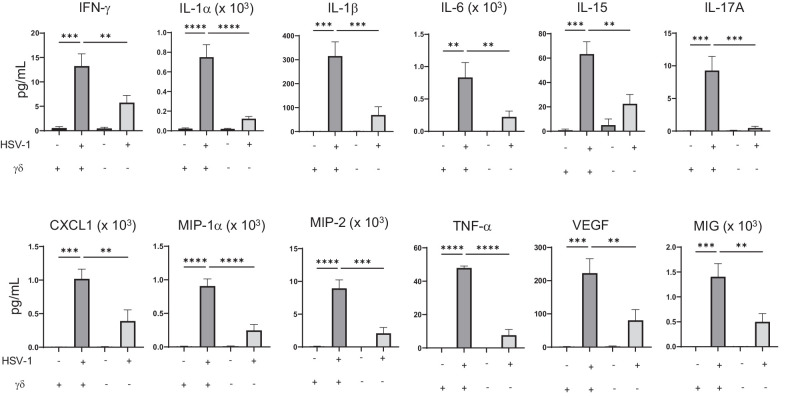
Cytokines and chemokines from infected and non-infected WT and TCRδ^−/−^ mouse corneas. Levels (pg/mL) of analytes in corneas from non-infected and 48-hour infected WT and TCRδ^−/−^ mice as determined by multiplex assay are shown (*n* = 3 to 6; corneas from two mice were pooled for each sample, and data are summarized from three independent experiments). **P < 0.007, ***P < 0.0008, ****P < 0.0001, one-way ANOVA, compared by Fisher's least significant difference (LSD) test.

To determine whether the corneal microenvironment is altered in the absence of γδ T cells, cytokine and chemokine production was compared in corneas from non-infected and HSV-1–infected WT and TCRδ^−/−^ mice. Non-infected corneas from both WT and TCRδ^−/−^ mice showed little or no cytokine or chemokine production (Fig. 2). Consistent with previous observations,^9^ levels of IL-17A were reduced in corneas from infected TCRδ^−/−^ mice compared to WT mice (Fig. 2). Not surprisingly, cytokines and chemokines known to be induced by IL-17A, such as IL-6, TNF-α, and CXCL1 were lower in corneas from infected TCRδ^−/−^ mice compared to WT mice (Fig. 2).^26^ Furthermore, IFN-γ and IL-15 were also reduced in infected corneas from TCRδ^−/−^ mice compared to those from WT mice. Because IL-15 regulates NK cell activity^27^ and NK cells are the primary producers of IFN-γ in infected corneas, these data suggest that NK cells are functionally impaired in TCRδ^−/−^ mice. Moreover, TCRδ^−/−^ mice had lower levels of MIG, an IFN-γ–stimulated chemokine,^28^^,^^29^ consistent with the reduction in NK cell–mediated IFN-γ in TCRδ^−/−^ mice.

### Altered Corneal NK Cell Gene Expression in Infected TCRδ^−/−^ Hosts

**Figure 3.**
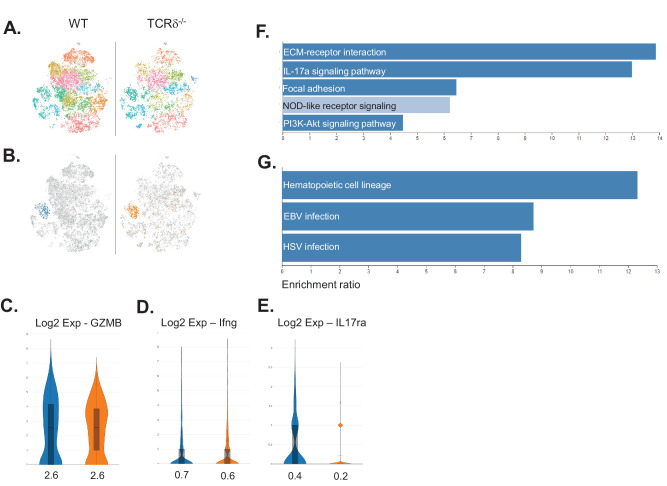
scRNAseq of corneas from WT and TCRδ^−/−^ mice at 48 hours PI. (**A**) t-SNE plots representing WT and TCRδ^−/−^ corneas. (**B**) NK cells from the WT (*blue*) and TCRδ^−/−^ (*orange*) corneas are overlaid on the t-SNE plot. (**C**–**E**) The log_2_ fold expressions of GZMB (**C**), IFN-γ (**D**), and IL-17A receptor (**E**) are shown; mean values are indicated on the *x*-axis. (**F**, **G**) KEGG pathway analysis of NK cells using the top 100 differentially expressed genes (DEGs) from WT (**F**) and TCRδ^−/−^ (**G**) mice; all *dark blue bars* had a false discovery rate (FDR) < 0.05.

scRNAseq was used to determine whether NK cell gene expression differs between WT and TCRδ^−/−^ HSV-1–infected corneas (Fig. 3A). NK cells were defined by the expression of *NKG7*, a gene specific for cytotoxic cells.^30^ NK cell frequency was comparable between both datasets, and NK cells appeared in the same cluster (Fig. 3B). Transcript levels of both GZMB and IFN-γ were comparable between NK cells from WT and TCRδ^−/−^ mice (Figs. 3C, 3D). This result was not surprising, given that both GZMB and IFN-γ can be tightly regulated posttranscriptionally.^31^^,^^32^ Interestingly, NK cells from WT corneas had a twofold higher expression of IL-17A receptor compared to those from TCRδ^−/−^ mice (Fig. 3E). Furthermore, KEGG pathway analysis revealed that NK cells from WT mice expressed genes involved in IL-17A signaling, whereas NK cells from TCRδ^−/−^ mice did not (Figs. 3F, 3G). In addition, the phosphoinositide 3-kinase (PI3K)/Akt signaling pathway was enriched in NK cells from WT mice, a pathway that is vital for NK cell activation,^33^ compared with NK cells from TCRδ^−/−^ mice (Fig. 3F). These data suggest that IL-17A produced by γδ T17 cells potentiates NK cell activation during the acute phase of ocular HSV-1 infection.

### TCRδ^−/−^ Mice Have Impaired Accumulation of Antiviral NK Cells

**Figure 4.**
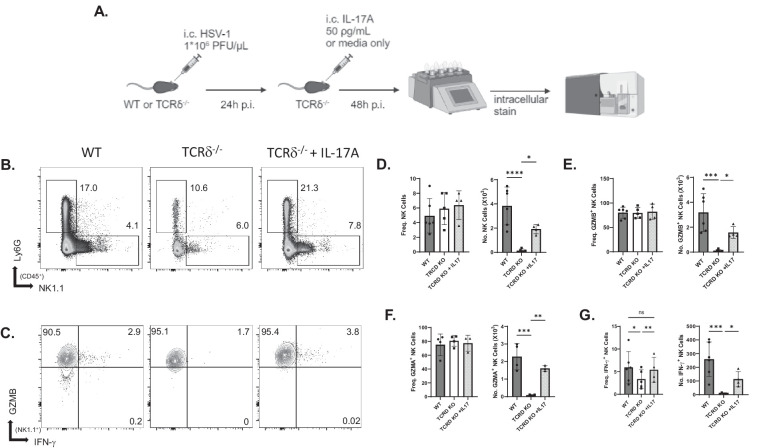
Effects of administration of IL-17A in TCRδ^−/−^ mice. (**A**) Schematic of the experimental approach for intracorneal (IC) HSV-1 infection and IL-17A administration of WT and TCRδ^−/−^ mice. (**B**) Representative flow cytometry plots of NK cells and neutrophils. (**C**) NK cells producing GZMB and IFN-γ. (**D**) Frequency (*left*) and number (*right*) of NK cells in WT, TCRδ^−/−^, and TCRδ^−/−^ + IL-17A corneas. (**E**–**G**) Frequency and number of GZMB-producing (**E**), GZMA-producing (**F**), and IFN-γ–producing (**G**) NK cells (*n* = 3 to 6; corneas from three to eight mice were pooled for each sample, and data are summarized from five independent experiments). *P < 0.05, **P < 0.007, ***P < 0.0002, ****P < 0.0001, one-way ANOVA compared by Fisher's LSD test.

To determine whether γδ T cells alter the activation and/or accumulation of NK cells in vivo, intracellular staining analysis by flow cytometry was performed on HSV-1–infected corneas (Fig. 4A). As an internal control, we analyzed the frequency and number of neutrophils as well as NK cells because it is known that TCRδ^−/−^ mice have impaired neutrophil chemotaxis (Fig. 4B). Although there was no difference in the frequency of NK cells producing GZMA or GZMB between WT and TCRδ^−/−^ mice, there was a significant increase in the number of granzyme-producing NK cells in WT corneas (Figs. 4C–F). IFN-γ–producing NK cells were significantly increased in both frequency and number in WT mice (Fig. 4G). These data demonstrate that γδ T cells are necessary for efficient accumulation of antiviral and granzyme-producing NK cells.

### Administration of IL-17A Rescues NK Cell Accumulation

IL-17A was administered to TCRδ^−/−^ mice to determine whether this γδ T17-cell–produced cytokine would restore NK cell accumulation in the infected cornea (Fig. 4A). Single administration of IL-17A at 24 hours PI increased the number of granzyme- and IFN-γ–producing NK cells (Figs. 4B–G). The frequency of IFN-γ–producing NK cells was also increased following IL-17A treatment (Fig. 4G). Consistent with IL-17A being necessary for neutrophil chemotaxis, the number of neutrophils was also restored in TCRδ^−/−^ mice given IL-17A (Fig. 4B). These results suggest that innate IL-17A produced by γδ T17 cells is required for optimal accumulation of granzyme- and IFN-γ–producing NK cells, as well as neutrophils.

**Figure 5.**
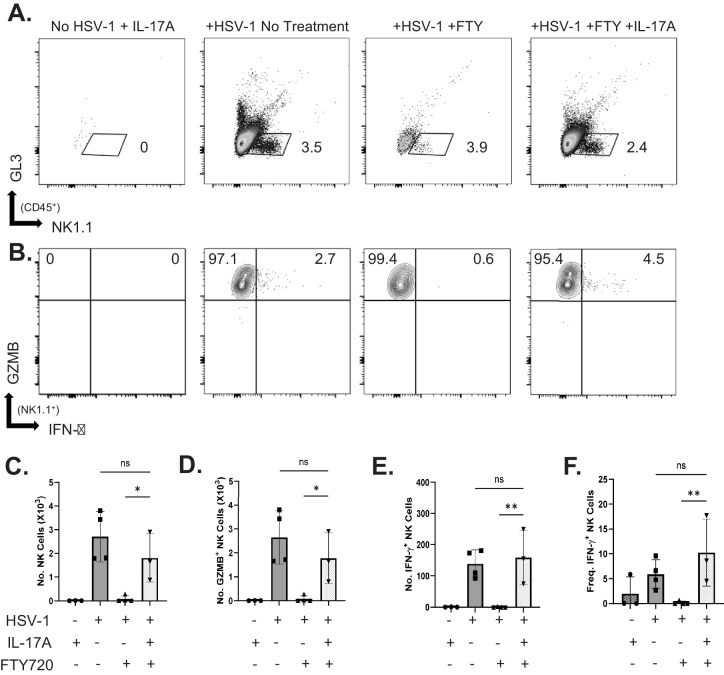
NK cell responses in WT mice treated with FTY720 ± IL-17A. (**A**, **B**) Shown is representative gating for NK cells (**A**) and GZMB-producing and IFN-γ–producing (**B**) NK cells. (**C**–**F**) Shown are the total NK cell number (**C**), number of GZMB-producing NK cells (**D**), number of IFN-γ–producing NK cells (**E**), and the frequency of IFN-γ–producing NK cells (**F**) (*n* = 3; corneas from three to five mice were pooled for each sample, and data are summarized from three independent experiments). *P < 0.02, **P < 0.006, two-way ANOVA with Tukey's correction (**C**, **D**, **E**) or Fisher's LSD test (**F**).

To test whether γδ T17 cells were specifically responsible for mediating the accumulation of antiviral NK cells in the HSV-1–infected cornea, WT mice were treated with FTY720. Importantly, FTY720 administration specifically reduced IL-17–producing CCR6^+^Vγ4^+^ γδ T-cell influx into the virus-infected corneas of WT mice (Supplementary Fig. S2). Infected WT mice receiving FTY720 also exhibited a significant reduction in NK cell influx compared to infected, untreated WT mice (Figs. 5A–C). Additionally, there were fewer granzyme and IFN-γ–producing NK cells in FTY720-treated hosts (Figs. 5D–F). To test whether IL-17A alone could restore NK cell accumulation, infected mice treated with FTY720 were simultaneously given IL-17A intracorneally. Administration of this cytokine completely restored granzyme-producing (Fig. 5D) and antiviral (Figs. 5E, 5F) NK cell influx. These results further support that IL-17A produced by γδ T17 cells is necessary for NK cell accumulation in the HSV-1–infected cornea.

### IL-17A Neutralization Reduces Accumulation of Protective NK Cells and Increases Viral Burden

**Figure 6.**
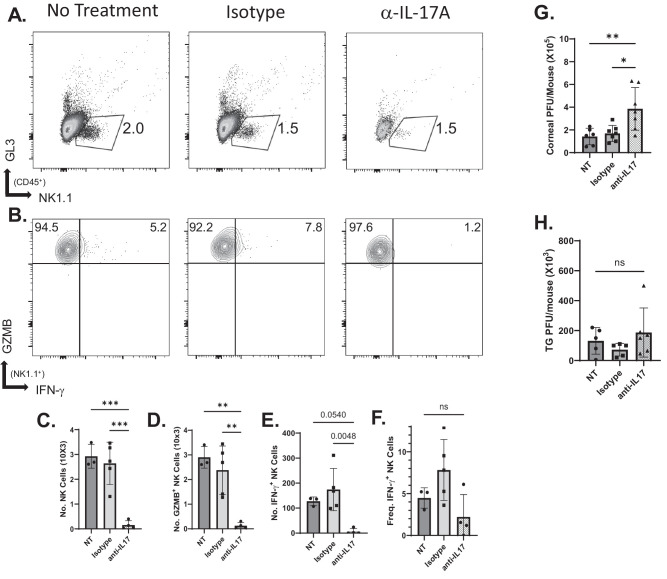
Effects of neutralizing IL-17A in WT mice. (**A**, **B**) Representative flow cytometry plots of NK cells (**A**) and GZMB-producing and IFN-γ–producing (**B**) NK cells from corneas 48 hours PI. (**C**–**E**) NK cell number (**C**) and number of GZMB-producing (**D**) and IFN-γ–producing (**E**) NK cells. (**F**) Frequency of IFN-γ–producing NK cells. (**G**, **H**) Viral burden in the cornea was determined by plaque assay (**G**), as well as by viral spread to the TG (**H**). Data shown are summarized from three independent experiments (*n* = 3; corneas from three to five mice were pooled for each sample). *P < 0.03, **P < 0.008, ***P < 0.0001, two-way ANOVA with Tukey's correction (**C**, **D**, **E**), and Fisher's LSD test (**F**, **H**).

If, in fact IL-17A production by γδ T17 cells is required for the NK cell response, we reasoned that neutralizing IL-17A in HSV-1–infected WT mice should also reduce the NK cell response. To test this, WT mice were given neutralizing IL-17A antibody or isotype control antibody or were left untreated at the time of infection. In mice receiving neutralizing IL-17A antibody, there was a significant reduction in the number of granzyme and IFN-γ–producing NK cells (Figs. 6A–F). We further tested whether antibody treatment affected viral growth and spread. At 48 hours PI, corneas from anti-IL-17A treated hosts had significantly higher viral titers compared to those in controls (Fig. 6G). HSV-1 viral titers did not differ from controls in the TG of anti-IL-17A receiving mice (Fig. 6H). Taken together, these results demonstrate the important role of γδ T17 cell production of IL-17A in protecting the host from early viral infection.

### IFN-γ Negatively Regulates Early IL-17A Production

**Figure 7.**
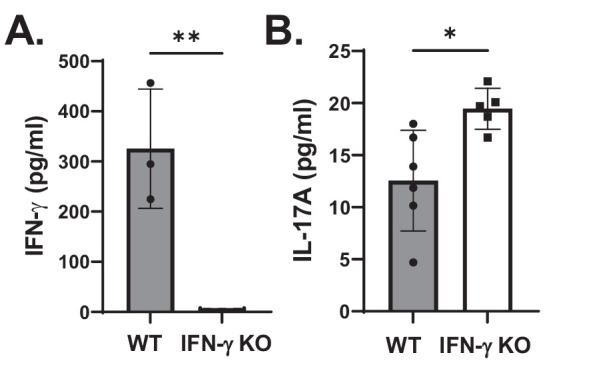
IFN-γ and IL-17A levels in the corneas of HSV-1–infected WT and IFN-γ^−/−^ mice. (**A**, **B**) Levels of IFN-γ (**A**) and IL-17A (**B**) as determined by ELISA 24 hours PI. Data shown are summarized from three independent experiments (*n* = 3 to 6 mice). *P < 0.02, **P < 0.001, unpaired *t*-test.

IFN-γ is primarily produced by NK cells in the acute phase of ocular HSV-1 infection.^9^ To determine whether NK cells may cross-regulate γδ T17 cells via production of IFN-γ, we tested whether IL-17A production in the cornea differed in corneas from HSV-1–infected WT and IFN-γ^−/−^ mice. Figure 7 shows that corneal IL-17A levels in IFN-γ^−/−^ mice were significantly increased compared to those measured in WT mice. As expected, IFN-γ was not detected in IFN-γ^−/−^ mice, whereas this cytokine was readily detected in WT mice. These data suggest that IFN-γ may limit IL-17A production in the virus infected cornea.

## Discussion

γδ T17 cells are critical for protection against ocular HSV-1 infection.^9^ They potentiate neutrophil chemotaxis to HSV-1–infected corneas,^12^ but whether and how γδ T cells regulate other innate immune responses, such as antiviral killing of HSV-1–infected cells, is unclear. Herein, we report that γδ T17 cells promote the recruitment and antiviral action of NK cells during ocular HSV-1 infection. Thus, NK cell migration is significantly reduced in HSV-1–infected corneas in mice lacking γδ T cells compared to WT mice. NK cell influx in corneas of TCRδ^−/−^ or FTY720-treated WT mice is rescued upon administration of IL-17A. Moreover, neutralization of IL-17A in WT mice was associated with enhanced viral titers in the cornea. Taken together, our data support a model whereby γδ T17-cell–produced IL-17A mediates antiviral NK cell responses in the infected cornea.

It is well established that IL-17A is necessary for neutrophil chemotaxis, both in the cornea and in other tissues.^7^^,^^12^^,^^34^^–^^36^. Our chemokine multiplex analysis revealed that several chemokines associated with neutrophil chemotaxis and activity (e.g., CXCL5, CXCL1, MIP-2, G-CSF, GM-CSF) were elevated in corneas from infected WT mice compared to those from TCRδ^−/−^ mice. Of this group, chemokines MIP-2 and CXCL5 were previously shown to be vital for neutrophil recruitment.^37^^,^^38^ It is known that IL-1α and, to a lesser extent, IL-1β are required to produce MIP-2, whereas CXCL5 is IL-1α dependent and TNF-α dependent.^37^^,^^38^ In a *Staphylococcus aureus* skin infection model, IL-17A/F^−/−^ mice had reduced IL-1α, IL-1β, and TNF-α,^39^ suggesting that IL-17 regulates these proinflammatory mediators. This finding is consistent with our observations that IL-1α, IL-1β, and TNF-α are reduced in HSV-1–infected corneas from TCRδ^−/−^ mice. Our results suggest that cytokines important for neutrophil recruitment to the cornea are regulated by IL-17A produced by γδ T cells. Neutrophils participating in the innate immune response can be pathogenic during ocular HSV-1 infection.^4^^,^^7^^,^^14^ Our data are consistent with a model whereby IL-17A–dependent regulation of neutrophil recruitment via chemokine production controls subsequent corneal damage.

NK cells are the predominant source of IFN-γ during the acute phase of ocular HSV-1 infection.^9^ Levels of IFN-γ, although comparable between corneas of WT and TCRδ^−/−^ mice at 24 hours PI,^9^ increased in corneas from WT mice at 48 hours PI, a time point when NK cells are most numerous. Reduced IFN-γ production in corneas from infected TCRδ^−/−^ mice correlated with a reduction in MIG. MIG is a chemokine induced by IFN-γ–mediated Janus kinase (JAK)/signal transducer and activator of transcription (STAT) signaling^29^ and may serve as a marker for active IFN-γ signaling.^28^ Thus, the reduction of MIG is consistent with reduced NK cell production of IFN-γ in TCRδ^−/−^ mice. Interestingly, levels of IL-15 were also approximately threefold lower in corneas from TCRδ^−/−^ mice compared to corneas from WT mice. IL-15 is a critical cytokine for the development and activation of NK cells.^27^ At the mRNA level, we also detected more IL-15 transcripts in neutrophils and monocytes from WT mice (data not shown). Whereas IL-15 mRNA is expressed by several cell types, IL-15 protein is mainly produced by activated monocytes, macrophages, and dendritic cells.^40^^,^^41^ Collectively, these data suggest that γδ T cells orchestrate chemotaxis and activation of a variety of innate immune cells, in addition to NK cells.

scRNAseq technology offers an unparalleled snapshot that can reveal complex heterogeneity between different physiologic settings,^42^ and it provides functional insights. We utilized this technology to generate the first scRNAseq library from HSV-1–infected corneas to determine whether NK cells were functionally impaired in the absence of γδ T cells. Differential gene expression analysis of NK cells from WT versus TCRδ^−/−^ mice revealed that PI3K/Akt signaling is enriched in WT NK cells. PI3K/Akt signaling is necessary for maturation and lytic functions of NK cells.^27^^,^^33^ Notably, PI3K/Akt signaling is downstream of IL-15–mediated NK cell activation^43^ and is necessary for efficient antiviral responses.^27^ Taken together with our multiplex analysis, our results support that γδ T cells are critical for IL-15–mediated activation of NK cells.

It is known that subsets of NK cells can respond to IL-17A in various pathogenic contexts.^15^^,^^44^^,^^45^ For example, in a preeclampsia model, IL-17A neutralization blunted NK cell cytotoxicity.^44^ Additionally, NK cell–mediated cytotoxicity and IFN-γ production were diminished in IL-17ra^−/−^ mice in response to *Candida albicans* infection.^15^ Although Bar et al.^15^ concluded that NK cell IL-17A signaling is important for NK cell development and less important for mediating NK cell function, our data suggest that direct IL-17A signaling is important for both accumulation and activation of NK cells during HSV-1 infection. Interestingly, scRNAseq analysis showed that IL-17RA expression was twofold higher in NK cells from corneas of WT compared to TCRδ^−/−^ mice. Moreover, pathway analysis revealed that IL-17A signaling was enriched in NK cells from WT corneas. Future experiments are required to determine whether IL-17A signaling directly or indirectly promotes NK cell activation.

IL-17A regulation of IFN-γ production appears to be context dependent. During infection, IL-17A can positively regulate IFN-γ production, although the mechanism is unknown.^46^^–^^49^ In other pathogenic models, the inverse is true. For example, in a model of visceral leishmaniasis, IL-17^−/−^ animals had enhanced IFN-γ production.^50^ The latter study focused on the αβ T-cell response or later time points after infection or treatment when the adaptive immune response is ongoing. Our findings strongly suggest that γδ T17 cells are necessary for NK cell antiviral activity during acute corneal HSV-1 infection. Thus, (1) administration of IL-17A to corneas of HSV-1–infected TCRδ^−/−^ mice rescues NK cell accumulation, (2) neutralization of IL-17A in WT mice impairs NK cell recruitment in the cornea and reduces viral clearance, and (3) inhibition of γδ T17 cell migration using FTY720 reduces NK cell influx. Consistent with our ocular HSV-1 infection model, γδ T cells were necessary for the accumulation of NK cells and neutrophils in a tracheal influenza infection study.^51^ Thus, depletion of γδ T cells led to reduced NK cells and viral clearance, increased weight loss, and higher mortality following influenza infection. However, in contrast to our results, in the influenza–tracheal infection model, depletion of the IL-17A–producing Vγ4^+^CCR6^+^ γδ T-cell subset did not affect accumulation of NK cells. In sum, our results support a model whereby γδ T17 cells are required for optimal NK cell accumulation in the cornea.

We used FTY720 to block γδ T17 cell migration following ocular HSV-1 infection. FTY720 blocks sphingosine-1-phospate receptor 1 (S1PR1), impairing lymphocyte migration to peripheral tissues.^52^ Treatment with FTY720 resulted in a marked reduction of IL-17A–producing γδ T cells in the skin of TLR7/8 agonist–treated mice.^53^ Because γδ T cells can be tissue resident,^54^ we needed to determine whether corneal γδ T17 cells were S1PR1 dependent during HSV-1 ocular infection. We found that γδ T17-cell migration was required, suggesting that γδ T17 cells do not constitute a corneal-resident population. Because FTY720 treatment effectively blocks both skin and corneal γδ T-cell migration, this would suggest a common mechanistic link between skin and corneal γδ T cells in their dependency for S1PR1.

Whether NK cells regulate γδ T cells is an understudied question. In a *Listeria* infection model, NK cell depletion resulted in increased γδ T cells in the peritoneum at day 5 PI.^55^ This result is consistent with our observations, using IFN-γ^−/−^ mice, that IFN-γ production by NK cells negatively regulates IL-17A production by γδ T17 cells during intracorneal infection. We previously observed that corneal IL-17A levels were similar between HSV-1–infected WT and NK cell-depleted mice.^9^ This differs from our result in this report indicating that IFN-γ^−/−^ mice produce significantly more IL-17A after HSV-1 infection. Importantly, NK cell-depleted mice still produced 0.8 pg/mL of IFN-γ,^9^ suggesting that anti-NK1.1 antibody treatment was not sufficient for complete NK cell depletion. Collectively, these results suggest to us that γδ T17 cells are sensitive to IFN-γ even at very low levels.

In summary, we report that γδ T17 cells positively regulate NK cell antiviral activity in the HSV-1–infected cornea. γδ T17 cells produce IL-17A following HSV-1 infection, which in turn mediates the accumulation of activated NK cells in the cornea. Conversely, NK cell production of IFN-γ appears to negatively regulate IL-17A production. Taken together, these results are consistent with a conceptual model whereby γδ T17 cells promote NK cell migration, which in turn regulates IL-17A production during acute infection (Supplementary Fig. S3). Because IL-17A can be pathogenic in large amounts in the cornea, this novel regulation may explain why and how γδ T17 cells do not cause corneal damage often associated with Th17 cells.

## Supplementary Material

Supplement 1

Supplement 2

Supplement 3
