# Supplementary material for: γδ T17 Cells Regulate the Acute Antiviral Response of NK Cells in HSV-1–Infected Corneas
Source: Invest Ophthalmol Vis Sci. 2024 Nov 6;65(13):16. doi: 10.1167/iovs.65.13.16 (PMC11549926; doi:10.1167/iovs.65.13.16)
Supplement: Supplement 1 [file iovs-65-13-16_s001.pdf]

A.

Quality Control Measurement	No. Events
Pre-Normalization Total No. of Reads	654,986,277
Post-Normalization Total No. of Reads	508,325,986
Pre-Normalization Mean Reads per Cell	29,311
Post-Normalization Mean Reads per Cell	22,748
Fraction of Reads Kept (WT)	65.6%
Fraction of Reads Kept (Tcrd KO)	100.0%
Pre-Normalization Total Reads per Cell (WT)	32,736
Pre-Normalization Total Reads per Cell (Tcrd KO)	24,526
Pre-Normalization Confidently Mapped Barcoded Reads per Cell (WT)	22,007
Pre-Normalization Confidently Mapped Barcoded Reads per Cell (Tcrd KO)	14,437
Estimated No. of Cells	22,346
Fraction Reads in Cells	87.6%
Median UMI Counts per Cell	5,042
Median Genes per Cell	1,780

B.

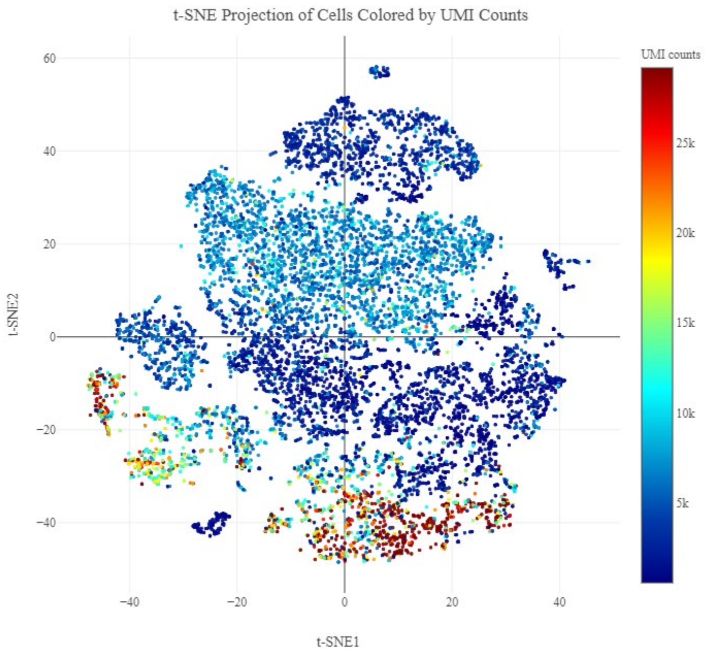

C.

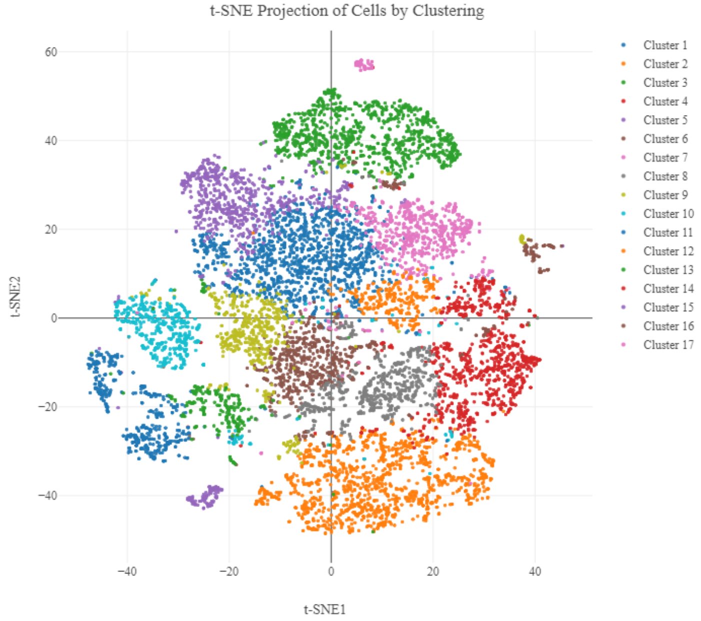

Supplemental Figure 1. Assessment of single cell RNA sequencing libraries. In (A), table examining cell counts, UMI counts per cell, and genes detected per cell. t-SNE projection of cells colored by UMI counts (B) and by clustering (C).
