# Supplementary material for: γδ T17 Cells Regulate the Acute Antiviral Response of NK Cells in HSV-1–Infected Corneas
Source: Invest Ophthalmol Vis Sci. 2024 Nov 6;65(13):16. doi: 10.1167/iovs.65.13.16 (PMC11549926; doi:10.1167/iovs.65.13.16)
Supplement: Supplement 2 [file iovs-65-13-16_s002.pdf]

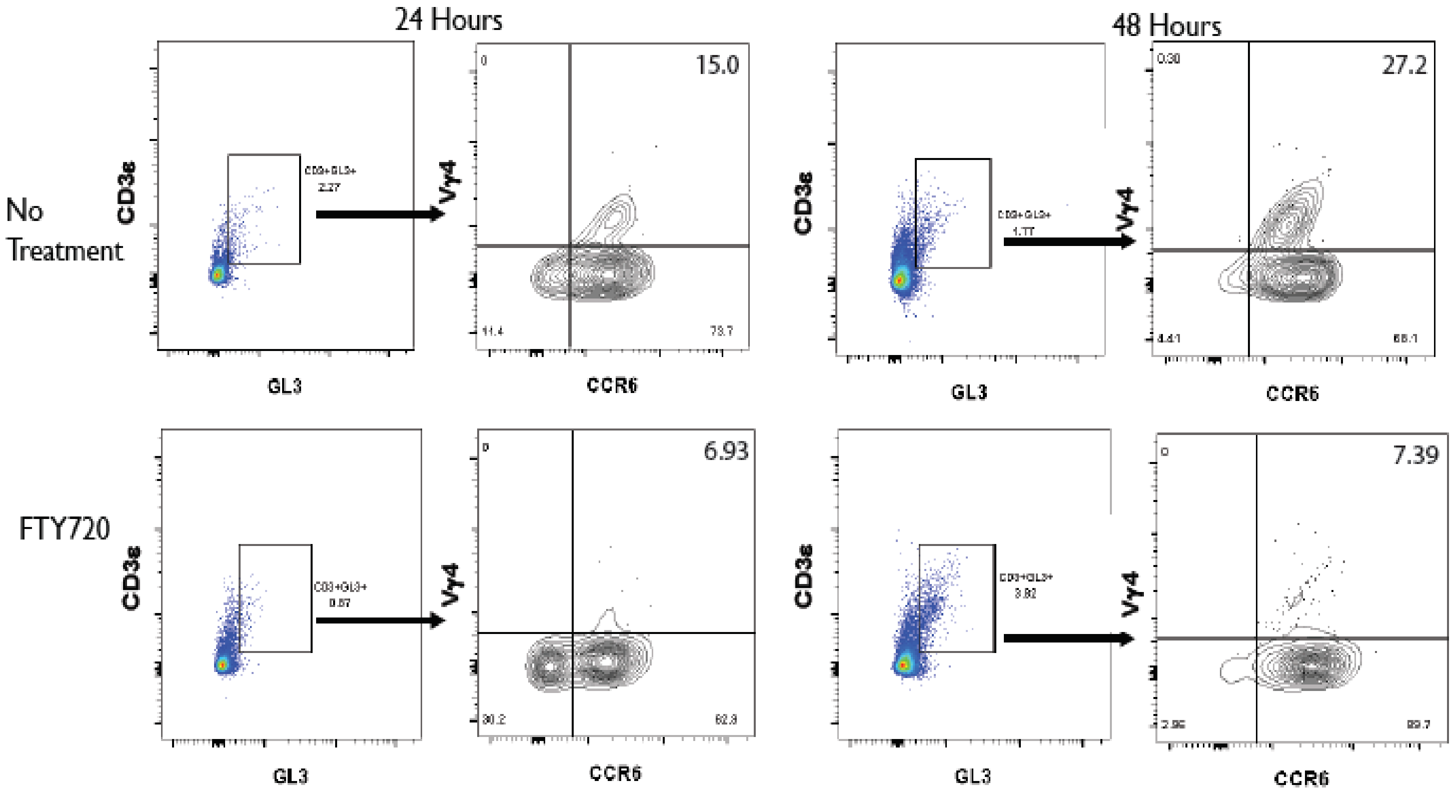

Supplemental Figure 2. Effect of FTY720 treatment on γδ T17 cells following corneal HSV-1 infection. Mice received either no treatment or FTY720 i.p. at 1 mg/kg. At 24 and 48h p.i., the frequency of $CCR6^{+}V\gamma4^{+}$ γδ T cells was determined by flow cytometry. By 48h p.i., the frequency of $CCR6^{+}V\gamma4^{+}$ γδ T cells is reduced in FTY720 treated mice (27.2% to 7.39%); *n*=2, corneas from 2 mice were pooled; data are representative from two experiments.
