# Supplementary material for: γδ T17 Cells Regulate the Acute Antiviral Response of NK Cells in HSV-1–Infected Corneas
Source: Invest Ophthalmol Vis Sci. 2024 Nov 6;65(13):16. doi: 10.1167/iovs.65.13.16 (PMC11549926; doi:10.1167/iovs.65.13.16)
Supplement: Supplement 3 [file iovs-65-13-16_s003.pdf]

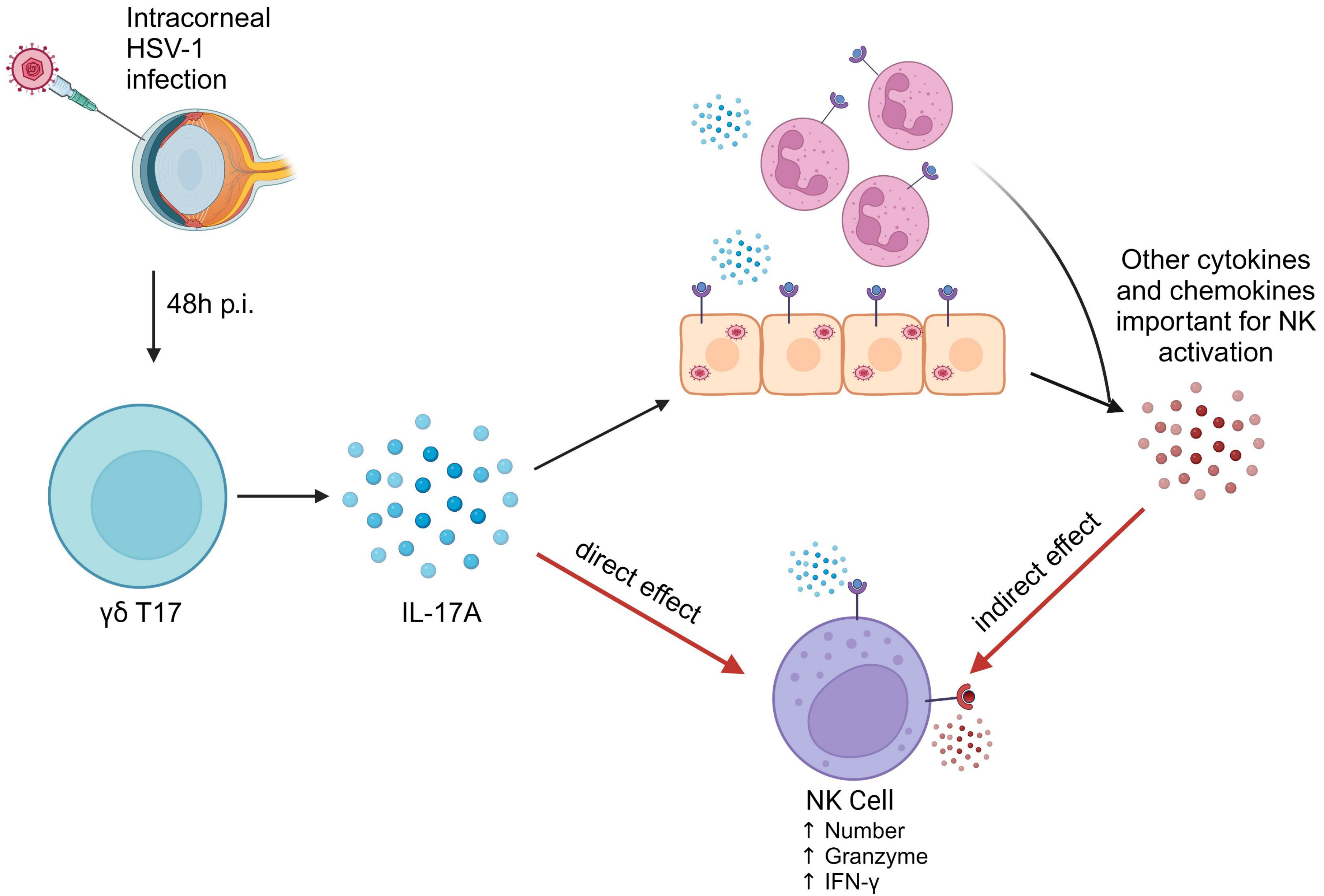

Supplemental Figure 3. Proposed mechanism for γδ T17 cell regulation of NK cells during HSV-1 corneal infection. Secreted IL-17A by recruited corneal γδ T17 cells acts directly and indirectly on NK cells. Indirect activity of IL-17A results from signaling through innate immune cells, fibroblasts and epithelial cells, stimulating the production of other cytokines and chemokines important for NK cell activation/accumulation.
